# Comparison of antegrade continence enema treatment and sacral nerve stimulation for children with severe functional constipation and fecal incontinence

**DOI:** 10.1111/nmo.13809

**Published:** 2020-02-03

**Authors:** Mana H. Vriesman, Lyon Wang, Candice Park, Karen A. Diefenbach, Marc A. Levitt, Richard J. Wood, Seth A. Alpert, Marc A. Benninga, Karla Vaz, Desale Yacob, Carlo Di Lorenzo, Peter L. Lu

**Affiliations:** ^1^ Division of Gastroenterology, Hepatology and Nutrition Department of Pediatrics Nationwide Children's Hospital Columbus OH USA; ^2^ Department of Pediatric Gastroenterology and Nutrition Emma Children's Hospital Amsterdam UMC University of Amsterdam Amsterdam The Netherlands; ^3^ The Ohio State University College of Medicine Columbus OH USA; ^4^ Department of Surgery Nationwide Children's Hospital Columbus OH USA; ^5^ Department of Urology Nationwide Children's Hospital Columbus OH USA

**Keywords:** antegrade continence enemas, children, functional constipation, neuromodulation, sacral nerve stimulation

## Abstract

**Background:**

To compare antegrade continence enema (ACE) treatment and sacral nerve stimulation (SNS) in children with intractable functional constipation (FC) and fecal incontinence (FI).

**Methods:**

We performed a retrospective review of children 6‐18 years old with FC and FI treated with either ACE or SNS at our institution. We recorded symptoms at baseline, 6 months, 12 months, 24 months, and their most recent visit after starting treatment. We compared improvement in FI, bowel movement (BM) frequency, abdominal pain, laxative use, and complications. Patients were contacted to evaluate perceived benefit using the Glasgow Children's Benefit Inventory.

**Key Results:**

We included 23 patients treated with ACE (52% female, median age 10 years) and 19 patients treated with SNS (74% female, median age 10 years). Improvement in FI was greater with SNS than ACE at 12 months (92.9% vs 57.1%, *P* = .03) and 24 months (100% vs 57.1%, *P* = .02). Improvement in BM frequency was greater with ACE, and children were more likely to discontinue laxatives at all follow‐up time points (all *P* < .05). Improvement in abdominal pain was greater with ACE at the most recent visit (*P* < .05). Rate of complications requiring surgery was similar between groups (26.3% vs 21.7%). Benefit was reported in 83.3% and 100% of ACE and SNS groups, respectively (NS).

**Conclusions and Inferences:**

Although both ACE and SNS can lead to durable improvement in children with FC and FI, SNS appears more effective for FI and ACE more effective in improving BM frequency and abdominal pain and in discontinuation of laxatives.

## Graphical abstract

Follow‐up data was available for 41 children at 6 months after treatment, 35 children at 12 months, 26 children at 24 months and all 42 children at most recent follow‐up. p‐value refers to difference in improvement from baseline. *p<0.05.

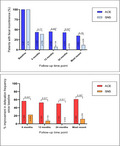

**Abbreviations**
ACE: antegrade continence enemas
ESPGHAN: European Society for Paediatric Gastroenterology Hepatology and Nutrition
FC: functional constipation
FI: fecal incontinence
GCBI: Glasgow Children's Benefit Inventory
NASPGHAN: North American Society for Pediatric Gastroenterology, Hepatology and Nutrition
SNS: sacral nerve stimulation

**Key Points**

- Antegrade continence enemas (ACE) and sacral nerve stimulation (SNS) have both been described as treatment options for children with intractable constipation.
- In our retrospective study, SNS led to greater improvement in FI, but ACE was more effective in improving bowel movement frequency, and decreasing laxative usage.
- Treatment of children with intractable constipation and FI should be individualized based on presentation. Larger, randomized studies are needed to better understand the roles of ACE and SNS.

## 1 INTRODUCTION

Functional constipation (FC) is a common condition in childhood with a described pooled prevalence of 9.5%.^1^ The diagnosis of FC is made through fulfillment of the Rome criteria.^2^ The majority of children with FC respond well to conventional medical and behavioral treatment strategies, including laxatives, dietary, and lifestyle changes.^3^ However, a sizable number of children remain symptomatic despite optimal conventional treatment and are considered to have intractable FC.^3^ Unfortunately, to date, treatment options for children with intractable FC are limited. According to the European Society for Paediatric Gastroenterology Hepatology and Nutrition (ESPGHAN) and North American Society for Pediatric Gastroenterology, Hepatology and Nutrition (NASPGHAN) guidelines on management of pediatric FC, transanal irrigation or surgical therapies including anal sphincter botulinum toxin injection, antegrade continence enemas (ACE), sacral nerve stimulation (SNS), and colonic resection could be considered in children with intractable FC.^3^ Generally, the decision for surgical management is based on a step‐up approach, beginning with the strategy that is least invasive and escalating accordingly as needed. Both ACE and SNS are considered minimally invasive surgical procedures.^3^, ^4^

Treatment with ACE involves antegrade flushing of colonic contents through a surgically created continent catheterizable channel in the abdominal wall. Since its introduction by Malone et al in 1990, modifications to the ACE procedure have led to well‐established laparoscopic techniques used for children with refractory constipation.^4^, ^5^ However, long‐term outcomes of ACE treatment for children with intractable FC have yet to be thoroughly clarified.

The relatively novel SNS treatment involves electric stimulation of the sacral nerve root by an implanted lead connected to a pulse generator battery and is thought to modulate the function of the bowel, bladder, and/or pelvic floor. Sacral nerve stimulation has been shown to be effective in treating both urinary and fecal incontinence (FI) in adults.^6^ However, the efficacy of SNS in the treatment of constipation is questionable^7^, ^8^ and experience with SNS in children remains very limited. A small number of studies have reported positive short‐ and long‐term outcomes for children with constipation and FI treated with SNS,^9^, ^10^, ^11^ but further research is needed.

The roles of ACE and SNS in the treatment of children with intractable FC therefore remain unclear, and guidelines for surgical treatment of intractable FC are lacking, leading to a wide variation in treatment practices between centers.^12^ No studies have yet compared outcomes of ACE and SNS. The objective of this study was to compare the efficacy and safety of ACE and SNS treatment for children with intractable FC and to assess perceived health‐related benefit and satisfaction.

## 2 MATERIALS AND METHODS

### 2.1 Study design and participants

This study consisted of two parts; first, we performed a retrospective cohort study comparing clinical symptoms and complications after starting ACE or SNS treatment. Next, we contacted all patients and parents included in the retrospective review to administer questionnaires assessing patient health‐related benefit and satisfaction. Our study protocol was approved by the local institutional review board.

We included children between 6 and 18 years with clinically confirmed FC and FI based on the Rome III criteria who were treated with either ACE or SNS at Nationwide Children's Hospital in Columbus, Ohio, USA from 2012 through 2016. Children with organic causes of constipation or with prior abdominal surgery were excluded. For all patients, the decision to proceed with either ACE or SNS was made by the treating physician and family.

### 2.2 Retrospective cohort study

Once we identified children meeting our inclusion and exclusion criteria, we recorded demographic information, medical and surgical history, and results of relevant diagnostic testing at baseline before ACE or SNS procedures. For each patient, information about clinical symptoms and complications was collected at 6 months, 12 months, 24 months, and at the most recent visit after starting ACE or SNS treatment. The most recent visit was defined as the latest follow‐up visit in the medical chart at which the patient was still receiving ACE or SNS treatment.

#### 2.2.1 Antegrade continence enema procedure

Children undergoing ACE treatment underwent either a Malone appendicostomy procedure or a percutaneous cecostomy procedure. Malone appendicostomy procedures were performed by a pediatric surgeon and involved connection of the appendix to the abdominal wall to create a valve for catheterization and ACE administration. Percutaneous cecostomy procedures were performed by an interventional radiologist and involved the percutaneous introduction of a cecostomy tube into the cecum for ACE administration. Specific ACE flush components were determined by the treating physician for each individual patient and generally consisted of a combination of normal saline or polyethylene glycol solution and a stimulant laxative (glycerin or bisacodyl).

#### 2.2.2 Sacral nerve stimulation procedure

The SNS procedures were performed by either a pediatric surgeon or pediatric urologist and were all done in two stages. The first stage involved placement of a lead at the S3 sacral nerve root connected to a temporary pulse generator that remained external to the patient for a 2‐week trial period. If clinical improvement was noted during this trial period, the patient underwent the second stage procedure. The second stage involved the implantation of a permanent pulse generator battery (InterStim® System, Medtronic, Inc) into the subcutaneous fat of the upper buttock. The distribution and amplitude of nerve stimulation was determined by the treating physician in order to achieve an effective and comfortable stimulation.

#### 2.2.3 Clinical outcomes

We compared improvement in FI frequency, bowel movement frequency, abdominal pain, and oral/rectal laxative use at each follow‐up time point for both treatment modalities. We defined successful treatment of FI as having FI less than once per week. We considered a bowel movement frequency of greater than twice per week as normal.^2^ We defined successful treatment of abdominal pain as having pain less than once per week. Improvement in laxative usage was defined as complete discontinuation of oral and rectal laxatives.

### 2.3 Patient benefit and satisfaction

To evaluate perceived patient benefit from each treatment, parents were contacted by telephone after the most recent follow‐up time point and asked to complete the Glasgow Children's Benefit Inventory (GCBI). The GCBI is a validated measure of health‐related benefit and contains 24 questions divided over four subscales (ie, “Emotion,” “Vitality,” “Learning,” and “Physical health”). Items are scored on a five‐point Likert scale ranging from “much worse” to “much better.” Total and subscale scores are then transposed to a benefit scale ranging from −100 (maximum harm) to +100 (maximum benefit). A GCBI score >0 indicates positive health‐related benefit.^12^ In order to assess patient satisfaction, we asked parents two questions: (a) Whether they would proceed with the treatment again if given the chance to remake their decision and (b) whether they would recommend the treatment to other families.

### 2.4 Statistical analysis

Chi‐square and Fisher's exact tests were used to compare outcomes of ACE vs SNS treatment at each follow‐up time point. Unpaired *t* tests and Mann‐Whitney *U* tests were performed for the comparison of numerical data. Patients were excluded from analyses if they stopped using ACE or SNS treatment or if they did not have a follow‐up visit within the time frame of interest. McNemar's test was used to compare the presence of symptoms at baseline to follow‐up within the same treatment group. A *P*‐value of <.05 was considered to be statistically significant. Statistical analyses were conducted using IBM SPSS version 24 (IBM, Armonk, NY).

## 3 RESULTS

**Figure 1:**
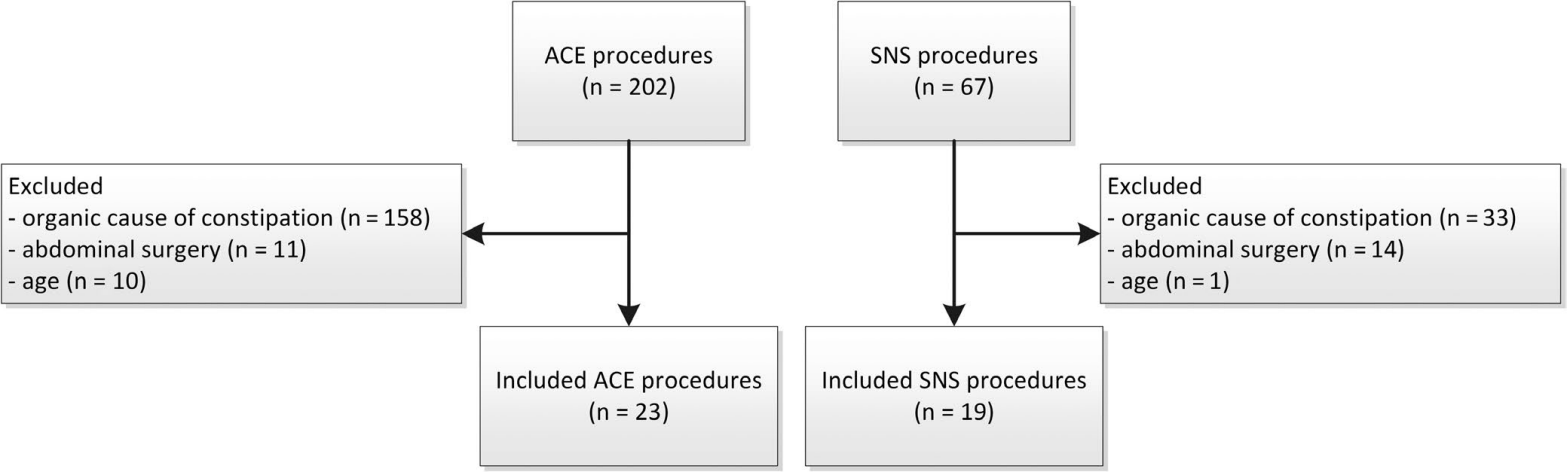
Flowchart inclusion and exclusion of patients

Of the 202 patients treated with ACE and 67 patients treated with SNS between 2012 and 2016, we included 23 patients treated with ACE (52.2% female, median age 10 years, range 6‐17) and 19 patients treated with SNS (73.7% female, median age 10 years at initiation, range 7‐16). Reasons for exclusion are depicted in Figure 1. All patients scheduled for the SNS procedure tolerated the 2‐week trial period and received a permanent pulse generator.

### 3.1 Retrospective cohort study

#### 3.1.1 Baseline characteristics

All patients fulfilled the Rome III criteria for FC and had symptoms for >12 months (mean 66 months) and were treated with oral laxatives before ACE or SNS treatment. More patients treated with ACE had been previously treated with rectal enemas compared to patients treated with SNS (87.0% vs 5.3%, *P* < .01). Patients treated with SNS were more likely to have concomitant urinary symptoms compared to patients treated with ACE (94.7% vs 30.4%, *P* < .01). Patient characteristics are shown in Table 1.

**Table 1:** Baseline patient characteristics

	Total (n = 42)	ACE group (n = 23)	SNS group (n = 19)	*P*‐value
Age, median (range)	10.3 (6.4‐17.4)	10.4 (6.4‐17.4)	10.2 (7.0‐16.4)	.46
Female, n (%)	26 (61.9)	12 (52.2)	14 (73.7)	.15
Procedure, n (%)				
‒ Cecostomy	–	22 (95.7)	–	–
‒ Malone		1 (4.3)		
Used oral laxatives, n (%)	42 (100)	23 (100)	19 (100)	1.0
Used rectal enemas, n (%)	21 (50.0)	20 (87.0)	1 (5.3)	**<.01** *
Duration of symptoms in months, mean (SD)	66.2 (36.9)	87.8 (34.2)	38.2 (15.3)	**<.01** *
Behavioral problems, n (%)	16 (38.1)	10 (43.5)	6 (31.6)	.43
Urinary incontinence, n (%)	25 (59.5)	7 (30.4)	18 (94.7)	**<.01** *

Note

–, not applicable.

Abbreviations: ACE, antegrade continence enema; SNS, sacral nerve stimulation.

* *P* < .05.

#### 3.1.2 Baseline diagnostic testing

Additional testing before the surgical procedure is displayed in Table 2. Significantly more children in the ACE group underwent additional diagnostic testing as compared to the patients treated with SNS (100% vs 73.7%, *P* < .01). One child in the ACE group with a prior normal colonic transit time also had a normal bowel movement frequency at baseline.

**Table 2:** Baseline diagnostic testing

	ACE (n = 23)	SNS (n = 19)	*P*‐value
Contrast enema, n (%)	22 (95.7%)	4 (21.1%)	**.00** *
‒ (Partial) redundant or distended colon	6 (27.3%)	2 (50.0%)	.56
Transit study, n (%)	13 (56.5%)	5 (26.3%)	**.05** *
‒ Delayed transit	4 (30.8%)	1 (20.0%)	.65
Anorectal manometry, n (%)	19 (82.6%)	11 (57.9%)	.08
‒ Dyssynergia	2 (10.5%)	1 (9.0%)	.70
Colonic manometry, n (%)	18 (78.3%)	1 (5.3%)	**.00** *
‒ (Partial) abnormal motility^a^	12 (66.7%)	0 (0.0%)	.18

Abbreviations: ACE, antegrade continence enema; SNS, sacral nerve stimulation.

a Premature termination of high‐amplitude propagating contractions in the colon.

* *P* < .05.

#### 3.1.3 Follow‐up

Follow‐up data were available for 41 children at 6 months (n = 23 ACE and n = 18 SNS, mean 5.6 months ± 1.6 SD) after treatment, 35 children at 12 months (n = 21 ACE and n = 14 SNS, mean 12.6 ± 2.2 SD), and 26 children at 24 months (n = 14 ACE and n = 12 SNS, mean 22.6 ± 2.4 SD) respectively. At 12 months after starting treatment, two patients had stopped using ACE treatment because their symptoms had improved. At 24 months after starting treatment, four more children (eg, total of six) had stopped using ACE because of clinical improvement and one patient stopped using SNS because the device had been removed due to a complication. The most recent visit of all 42 children was at a median of 22 months (range 3‐52) after treatment initiation.

#### 3.1.4 Fecal incontinence

**Figure 2:**
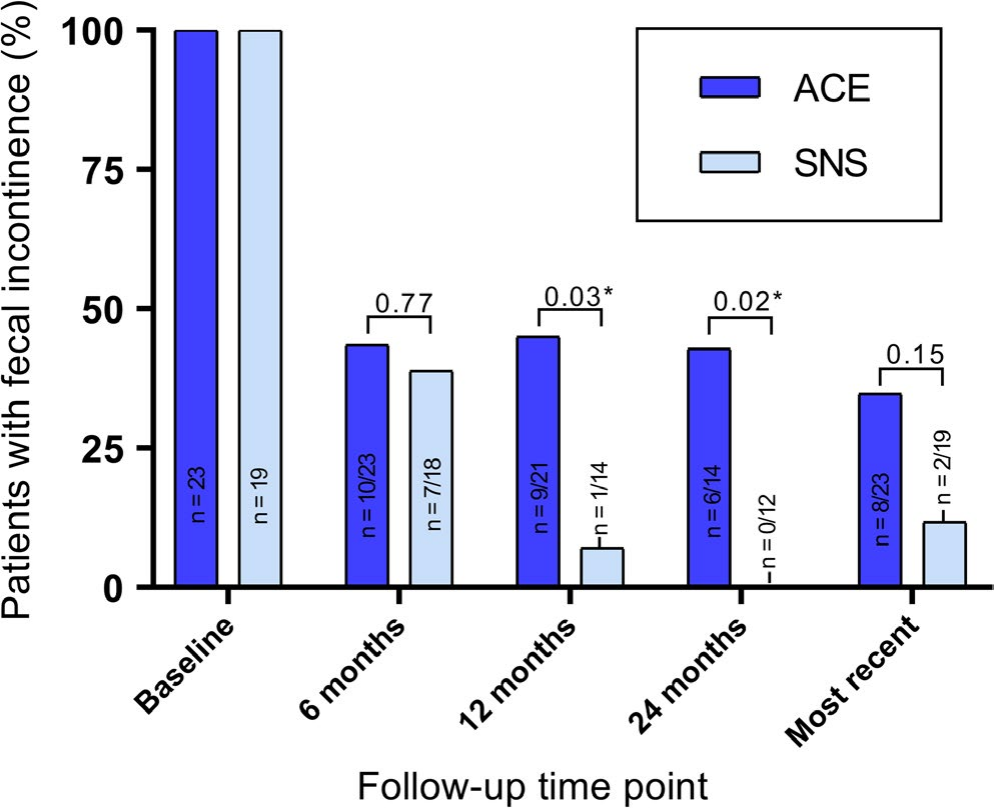
Patients with fecal incontinence at follow‐up. *P < .05. *P*‐value refers to difference in improvement from baseline. Follow‐up data were available for 41 children at 6 mo after treatment, 35 children at 12 mo, 26 children at 24 mo, and all 42 children at most recent follow‐up

Figure 2 shows the percentage of patients with FI at follow‐up time points. Compared with baseline, children in both ACE and SNS groups had significant improvement in FI by 6 months of treatment and this improvement remained significant at 12 months, 24 months, and at the most recent visit (all *P* < .05). Improvement of FI was significantly greater for patients treated with SNS than ACE at 12‐month (n = 13/14 [92.9%] vs n = 12/21 [57.1%], *P* = .03) and 24‐month follow‐up (n = 11/11 [100%] vs n = 8/14 [57.1%], *P* = .02). Out of the 42 patients with FI at baseline, two patients treated with SNS (11.8%) and eight patients treated with ACE (34.8%) still had symptoms of FI at the most recent visit (*P* = .15).

#### 3.1.5 Bowel movement frequency

**Figure 3:**
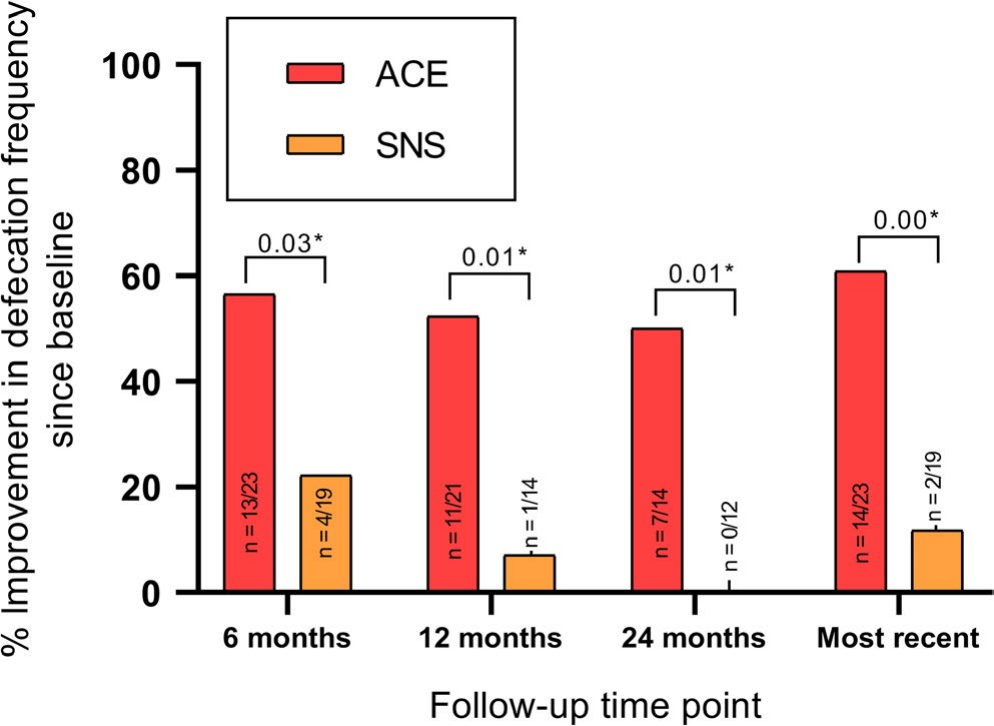
Patients with improvement in defecation frequency at follow‐up. *P < .05. *P*‐value refers to difference in improvement from baseline. Follow‐up data were available for 41 children at 6 mo after treatment, 35 children at 12 mo, 26 children at 24 mo, and all 42 children at most recent follow‐up

At baseline before starting ACE or SNS, 10/23 (43.5%) of the ACE patients had >2 bowel movements per week vs 15/19 (78.9%) of SNS patients (*P* = .02). At the most recent visit, 22/23 (95.7%) of ACE patients had >2 bowel movements per week vs 14/19 (82.4%) of SNS patients (*P* = .29). As shown in Figure 3, improvement in bowel movement frequency (ie, >2 per week) was significantly greater for patients treated with ACE than SNS at all follow‐up time points (all *P* < .05). In the ACE group, 22/23 (95.7%) children had a bowel movement frequency >2 per week at most recent follow‐up compared with 10/23 (43.5%) at baseline (*P* < .01). No significant improvement in bowel movement frequency was found for patients treated with SNS (*P* = 1.0) at the most recent follow‐up compared with baseline.

#### 3.1.6 Abdominal pain

As depicted in Table 3, we compared the number of patients with abdominal pain at baseline to all consecutive follow‐up time points (ie, 6 months, 12 months, 24 months and most recent visit). In the ACE group, fewer children had symptoms of abdominal pain at each follow‐up time point compared with baseline (all *P* < .05). In the SNS group, the number of children with abdominal pain did not differ significantly between baseline and the consecutive follow‐up time points. The number of patients with improvement of abdominal pain was greater for patients treated with ACE than SNS at the most recent visit (n = 10 [45.5%] vs n = 1 [7.7%], *P* < .05).

**Table 3:** Difference in clinical symptoms at follow‐up between SNS and ACE treatment group

	ACE		SNS		
	n (%)	% improvement since baseline	n (%)	% improvement since baseline	*P*‐value^a^
Abdominal pain					
Baseline	15/23 (65.2)	–	6/19 (31.5)	–	
6 mo	8/23 (34.8)	36.4%	6/14 (42.9)	15.4%	.23
12 mo	4/20 (20.0)	45.0%	4/12 (33.3)	20.0%	.25
24 mo	3/14 (21.4)	42.9%	5/9 (55.6)	0.0%	.06
Most recent	6/23 (26.1)	45.5%	6/19 (31.5)	7.7%	**.03** *
Use of oral/rectal laxatives					
Baseline	23/23 (100)	–	17/19 (89.5)	–	
6 mo	6/23 (26.1)	73.9%	15/18 (83.3)	5.6%	**.00** *
12 mo	3/20 (15.0)	81.0%	13/15 (86.7)	6.7%	**.00** *
24 mo	3/14 (21.4)	78.6%	8/12 (66.7)	16.7%	**.00** *
Most recent	8/23 (34.8)	52.2%	16/19 (84.2)	5.3%	**.00** *

a *P*‐value represents difference in % improvement since baseline.

* *P* < .05.

#### 3.1.7 Laxative treatment

At baseline, the majority of ACE and SNS patients were treated with laxatives (n = 23 [100%] vs n = 17 [89.5%], respectively). Patients treated with ACE were more likely to be able to discontinue oral and/or rectal laxative use at all follow‐up time points compared to patients treated with SNS (all *P* < .01, Table 3). At the most recent visit, 8 of 23 patients (34.8%) treated with ACE used oral laxatives compared to 16 of 19 patients (82.4%) treated with SNS (*P* < .01).

#### 3.1.8 Complications

Overall complications were more common in the ACE group compared with the SNS group (19/23 [82.6%] vs 5/19 [26.3%], *P* < .01). Of the 19 patients in the ACE group with a reported complication, 14 (73.6%) children had a minor complication (ie, granulation tissue or leakage of the cecostomy tube). The number of patients who had severe complications requiring further surgery was similar between ACE and SNS groups (n = 5/23 [21.7%] vs n = 5/19 [26.3%], *P* = 1.0). In the ACE group, five patients required further surgery: two required laparoscopic partial colonic resection and one required a diverting ileostomy due to treatment failure, one required a revision due to wound infection, and one required a revision due to significant leakage. In the SNS group, five patients required further surgery: three required device removal and replacement due to wound infection, one required a revision due to development of a fluid collection at the device that was causing leg discomfort, and one required device replacement due to stimulator malfunction. One of these five patients subsequently required Malone appendicostomy creation due to treatment failure.

### 3.2 Patient benefit and satisfaction

All 42 patients were contacted by telephone to fill out the GCBI and satisfaction questionnaires. The families of 12 of 23 (52.1%) patients in the ACE group and 9 of 19 (47.4%) patients in the SNS group completed the questionnaires over the phone. The remainder of the families were unable to be reached (10 patients treated with ACE and 9 treated with SNS) or unable to complete the questionnaire due to prior device removal (1 patient treated with ACE and 2 patients treated with SNS). Families completed questionnaires a median of 4.0 years after ACE initiation and 4.8 years after SNS initiation.

The median GCBI score was +45.8 (IQR 9.4‐67.7) for the ACE group and +43.8 (IQR 16.7‐87.5) for the SNS group (*P* = .67). Ten of 12 families (83.3%) in the ACE group reported positive health‐related benefit from the procedure compared with 9 of 9 (100%) in the SNS group (*P* = 1.0). Eleven of 12 families (91.6%) in the ACE group and 8 of 9 families (88.9%) in the SNS group indicated they would repeat the procedure if given the opportunity and would recommend it to others (*P* = 1.0). When asked to explain their answers, the ACE family who would not repeat or recommend the procedure commented that the indwelling tube made their child much more anxious. The SNS family commented that their child's symptoms did not improve significantly.

## 4 DISCUSSION

In this retrospective comparison of ACE and SNS treatment for children with intractable constipation, we found that both ACE and SNS led to durable improvement of FC and FI symptoms. However, the effects of ACE and SNS on specific symptoms were different. ACE led to greater improvement in bowel movement frequency and abdominal pain, while SNS led to greater improvement in FI, with a nearly 90% decrease in FI after 2 years of SNS treatment.

The improvement we observed in FI with SNS treatment is consistent with prior literature in adults with FI. Sacral nerve stimulation has been approved by the United States Food and Drug Administration (FDA) for adults with FI unresponsive to conventional therapy.^7^, ^13^ In the pediatric population, improvement of FI after SNS treatment has been described in small cohorts of children with dysfunctional elimination syndrome,^14^, ^15^ neurological conditions,^16^ bladder and bowel dysfunction^8^ and FC.^11^ ACE treatment led to significant improvement in FI in our cohort as well, although to a lesser degree than SNS. Higher success rates have been previously reported. In a recent study of long‐term outcomes of ACE treatment that included 93 children with FC, 86% of children no longer had FI after 26 months of ACE treatment.^17^ Variation in the use of ACE treatment (frequency of administration and composition of cleansing solution) may contribute to the differences observed.^18^

Although our findings support the ability of SNS treatment to decrease FI, they do not support its role in the improvement of defecation frequency. The majority of patients treated with SNS had normal bowel movement frequency at baseline; however, SNS did not improve defecation frequency beyond each child's baseline laxative regimen at follow‐up. Contradictory results have been published previously. A small study including 12 children with FC showed that 92% of children had a normal bowel movement frequency (eg, >2 a week) 6 months post‐SNS procedure.^9^ Another study reported a significant increase in bowel movement frequency after just 3 weeks of SNS treatment (5.9 vs 17.4, *P* < .01).^10^ However, a long‐term study at our institution showed no significant improvement in defecation frequency after more than 2 years of follow‐up.^11^ Randomized controlled trials in the adult population show similar negative results.^19^, ^20^ In contrast, in the ACE group, the number of children with a normal defecation frequency increased to 100% within the first 6 months. Long‐term follow‐up of patients after ACE showed similar high rates of improvement of symptoms up to 80%.^21^ Our results therefore support the use of ACE treatment for children with intractable FC.

The differences between the effects of ACE and SNS in treating FC and FI are potentially secondary to different pathways of action. Treatment with ACE works through mechanical irrigation of bowel contents, potentially in conjunction with stimulation of propagating colonic contractions, allowing the colon to fully evacuate on a regular basis and leads to improvement in FI by preventing stool accumulation. Although the precise mechanism by which SNS leads to improvement in FI remains incompletely understood, there is evidence that SNS modulates anorectal function, both centrally and peripherally.^22^ Studies in adults showed that SNS may affect colonic motility by increasing the frequency of both antegrade and retrograde propagating pressure waves in patients with slow‐transit constipation.^23^, ^24^ These effects on anorectal function or increased retrograde motor function in the colon may explain its ability to decrease FI.^22^, ^25^

The possibility remains that the described differences in clinical outcomes between ACE and SNS treatment may be in part secondary to selection bias. At the time of our study, no guidelines on the management of intractable constipation were available and the decision for either ACE or SNS was based on the clinical experience of the treating physician. Therefore, our results should be interpreted with care. Although we aimed for a homogenous population of children with intractable FC and FI, there were important differences in baseline characteristics between groups. Factors such as the presence of urinary symptoms and results from additional testing could have influenced the decision to use ACE or SNS and treatment outcome. Children treated with ACE at baseline had a longer duration of symptoms, fewer bowel movements per week and more frequent use of enemas as compared to the children treated with SNS. It could therefore also be hypothesized that children in the ACE group had more severe symptoms of constipation, negatively affecting treatment outcome. The possibility of selection bias is in part inherent to our retrospective study design, and future prospective studies with larger sample sizes and matched controls with a standardized protocol for diagnostic additional testing are needed to confirm our results.

A proportion of our patients had normal bowel frequency and transit time at baseline, raising the question of whether these children had non‐retentive fecal incontinence instead of FC. We verified that all patients fulfilled the Rome III criteria for FC at initial presentation and only one child with normal transit time also had a normal bowel movement frequency at baseline. Normal transit time itself does not contribute to the diagnosis of either non‐retentive fecal incontinence or FC without the combination of clinical symptoms.^3^, ^26^ Moreover, the normal bowel movement frequency at baseline in some children is likely explained by the fact that almost all patients were treated with oral and/or rectal laxatives before starting ACE or SNS.

Information about improvement in urinary symptoms could not be assessed since this was not consistently reported for our ACE patients in the medical record. Although SNS is considered effective in treating urinary symptoms,^16^, ^27^ it would be interesting to compare results between SNS and ACE in children with FC. Moreover, the use of Malone as an antegrade option for FC at our institution became more widely available during the time span of this study. Consequently, only one patient in our cohort received a Malone procedure and twenty‐two children with a percutaneous cecostomy procedure. Therefore, caution should be taking in mind when extrapolating our results to children with a Malone appendicostomy. However, our results on FI, bowel movement frequency and complications are in line with the previous reported literature on patients with a Malone appendecostomy.^28^

In order to further compare both treatment options, we also evaluated complications. Although both neurostimulation and ACE are considered minimally invasive surgical procedures,^3^, ^4^ both techniques require general anesthesia and can lead to potential complications and side effects. The complication rates in our cohort are similar to the previously described literature on both ACE and SNS in children.^14^, ^15^, ^16^, ^21^, ^28^, ^29^, ^30^ Although the prevalence of complications in the ACE group was higher as compared to the SNS group, most complications after ACE were considered minor and the number of patients that required further surgery was similar between the two groups (26% and 22%, respectively). All complications reported in the SNS group required a surgical revision. Therefore, although SNS could be considered less invasive than abdominal surgery required for ACE, it is important to keep in mind that patients undergoing SNS treatment require two surgical procedures with general anesthesia to start treatment and these procedures are only performed in a few experienced centers. Moreover, financial costs associated with SNS treatment can be substantial.^31^ We therefore stress that patients and their families should be educated about the possible risks of both treatments in order to make an informed decision.

Despite the associated complications and financial cost, families generally viewed ACE and SNS favorably. Although we were only able to contact half of our study population, we showed high patient perceived benefit and satisfaction after more than 4 years of treatment. More importantly, we found no significant differences in perceived benefit scores and satisfaction scores between children treated with ACE and SNS. These results support the use of both treatment strategies for children with severe FC and FI. However, owing to the small study sample and potential selection bias, prospective studies comparing quality of life, perceived benefit, and satisfaction after both surgeries are needed.

In conclusion, this retrospective comparison shows that both ACE and SNS treatments can be effective for children with intractable FC and FI. The ideal treatment option for each child should be based on his or her personal clinical symptoms. Our findings suggest that children with severe FI and concurrent urinary symptoms may benefit more from SNS treatment, while children who struggle primarily with stool evacuation and abdominal pain may benefit more from ACE treatment. Obviously, both ACE and SNS should only be considered in patients with severe symptoms refractory to conventional treatment. Although considered minimally invasive, both therapies require surgical procedures with risk of severe complications. Prospective randomized studies comparing outcomes after ACE and SNS in a larger, homogenous cohort of children with FC are needed to better understand the optimal treatment strategy for children with intractable FC and FI.

## DISCLOSURE

The authors have no financial relationships relevant to this article to disclose. The authors have no conflicts of interest to disclose.
